# Circulating Human Eosinophils Share a Similar Transcriptional Profile in Asthma and Other Hypereosinophilic Disorders

**DOI:** 10.1371/journal.pone.0141740

**Published:** 2015-11-02

**Authors:** Cindy Barnig, Ghada Alsaleh, Nicolas Jung, Doulaye Dembélé, Nicodème Paul, Anh Poirot, Béatrice Uring-Lambert, Philippe Georgel, Fréderic de Blay, Seiamak Bahram

**Affiliations:** 1 INSERM UMR S_1109, ImmunoRhumatologie Moléculaire, Labex Transplantex, Fédération de Médecine Translationnelle de Strasbourg, Université de Strasbourg, 67085, Strasbourg Cedex, France; 2 Service de Pneumologie, Hôpitaux Universitaires de Strasbourg, Nouvel Hôpital Civil, 67091, Strasbourg Cedex, France; 3 Fédération Hospitalo-Universitaire, OMICARE, Centre de Recherche d’Immunologie et d’Hématologie, 67085, Strasbourg, France; 4 IRMA, CNRS UMR 7501, Labex IRMIA, Université de Strasbourg, 67084, Strasbourg Cedex, France; 5 Institut de Génétique et de Biologie Moléculaire et Cellulaire, INSERM U964, CNRS UMR 7104, Université de Strasbourg, 67404, Illkirch, France; 6 Laboratoire Central d’Immunologie, Pôle de Biologie, Nouvel Hôpital Civil, Hôpitaux Universitaires de Strasbourg, 67091, Strasbourg Cedex, France; Jackson Laboratory, UNITED STATES

## Abstract

Eosinophils are leukocytes that are released into the peripheral blood in a phenotypically mature state and are capable of being recruited into tissues in response to appropriate stimuli. Eosinophils, traditionally considered cytotoxic effector cells, are leukocytes recruited into the airways of asthma patients where they are believed to contribute to the development of many features of the disease. This perception, however, has been challenged by recent findings suggesting that eosinophils have also immunomodulatory functions and may be involved in tissue homeostasis and wound healing. Here we describe a transcriptome-based approach–in a limited number of patients and controls—to investigate the activation state of circulating human eosinophils isolated by flow cytometry. We provide an overview of the global expression pattern in eosinophils in various relevant conditions, e.g., eosinophilic asthma, hypereosinophilic dermatological diseases, parasitosis and pulmonary aspergillosis. Compared to healthy subjects, circulating eosinophils isolated from asthma patients differed in their gene expression profile which is marked by downregulation of transcripts involved in antigen presentation, pathogen recognition and mucosal innate immunity, whereas up-regulated genes were involved in response to non-specific stimulation, wounding and maintenance of homeostasis. Eosinophils from other hypereosinophilic disorders displayed a very similar transcriptional profile. Taken together, these observations seem to indicate that eosinophils exhibit non-specific immunomodulatory functions important for tissue repair and homeostasis and suggest new roles for these cells in asthma immunobiology.

## Introduction

Eosinophils are bone marrow-derived leukocytes that are released into the peripheral blood in a phenotypically mature state where they represent less than 5% of the leukocyte population. They are capable of being recruited to tissues in response to appropriate stimuli. In healthy individuals, most eosinophils are found in the gut, mammary glands, uterus, thymus, bone marrow and adipose tissues [1] but their role in these tissues at baseline conditions, as well as their close interactions with mast cells, is still under investigation.

Mature human eosinophils contain crystalloid secondary granules, which are primarily composed of highly charged basic proteins including two eosinophil granule major basic proteins (MBPs), eosinophil cationic protein (ECP), eosinophil derived neurotoxin (EDN) and eosinophil peroxidase (EPO). MBP, EPO, and ECP are toxic to a variety of tissues, including the bronchial epithelium [1]. Eosinophils also express a broad range of cytokines; e.g. Th2 (such as IL-4, IL-5, IL-9, IL-13, and IL-25), Th1 (IL-12 and IFN-γ), acute pro-inflammatory cytokines (TNF-α, IL-1β, IL-6 and IL-8), immune inhibitory cytokines (e.g, TGF-β and IL-10); as well as receptors for many of these cytokines [1].

Eosinophils have been typically considered as end-stage destructive cells involved in host protection against parasites [2]. They are also recruited into the airways of asthma patients and the release of their granule proteins has been suggested to contribute to the development of many features of asthma, including airways hyperresponsiveness and mucus hypersecretion [3]. However, the development of different strategies over the last years targeting eosinophils and their effector functions in patients with asthma has been disappointing and only reducing exacerbations in a selected asthma population [4, 5]. Moreover data from genetically engineered strains of mice affecting eosinophil effector functions or eosinophils themselves have shown that eosinophils might be dispensable in allergic airway inflammation [6, 7].

The accumulation and increase in eosinophils are by no means unique to helminth infections and asthma, since blood and tissue eosinophilia is also encountered in numerous other disorders, such as atopic dermatitis, Churg-Strauss syndrome, various infections, adverse drug reactions, gastrointestinal disorders or certain malignancies [8]. Yet, their very role in such hypereosinophilic diseases remains, surprisingly, unclear.

Recent findings in mouse models support a broader role for eosinophils and suggest that they may be important in the regulation of the broader immune response. At baseline conditions, recent work has indeed shown that eosinophils can contribute to homeostatic processes, for example plasma cell maintenance in the bone marrow as well as metabolic homeostasis [9]. Other data indicate that these cells might also function as reparative cells during inflammatory responses to heal injured tissues and to promote homeostasis [10, 11].

The aim of our work was to compare the transcriptional profile of circulating eosinophils from asthma patients vs those from healthy controls and other hypereosinophilic diseases. Our undermining working hypothesis was to uncover a disease-specific expression profile of eosinophils in asthma versus healthy controls and/or other hypereosinophilic syndromes. Whole genome expression analyses remain a fairly unbiased approach in comparing global gene activity between different states of a cell, tissue, organ, organism. In case of eosinophils however, this approach has been hampered by the extreme difficulty to harvest these cells in sufficient amounts and in a “healthy” state in order to obtain high grade RNA [12]. Having overcome these technical hindrances, we managed to set-up a robust process for a transcriptome-based analysis of eosinophils. Using circulating cells isolated from a limited number of healthy donors and patients with eosinophilic asthma or other hypereosinophilic diseases, we report here the transcriptional pattern of these cells. Our data suggest a common modulation of gene expression in disease upon cell activation and provide additional insights supporting a broad role of eosinophils in tissue repair and homeostasis.

## Material and Methods

### Characteristics of subjects

Main characteristics of subjects are summarized in Table 1. Patients with asthma (n = 7) ranged in age from 27 to 68, had persistent, moderate-to-severe disease and elevated blood eosinophil count (> 500 x 10^9^/L). Five of them had also documented nasal polyposis. All asthmatics patients were on high doses of inhaled corticosteroids, none of the subjects with asthma was taking oral corticosteroids at the time of blood withdrawal. Subjects with peripheral hypereosinophilia defined as in [13] related to other diseases were also recruited, including subjects with dermatologic disease (n = 3, i.e prurigo, bullous pemphigoid and drug toxidermia), parasitosis (n = 3, i.e giardiasis, ascaridiosis and scabies) and pulmonary aspergillosis (n = 5, with 2 of them also suffering from asthma associated to bronchopulmonary aspergillosis.). Healthy non-atopic control subjects (n = 10) had peripheral eosinophil counts below 500 x 10^9^/L. Peripheral ethylenediamine tetra-acetic acid (EDTA) anticoagulated blood was collected by venipuncture (20 ml) as part of routine medical examination from subjects who gave their informed written consent to this Institutional Review Board (Comité de Protection des Personnes–EST; STRASBOURG) approved protocol.

**Table 1:** Subject characteristics.

	Control subjects	Asthma	Dermatological disease	Parasitosis	Pulmonary aspergillosis
***n***	10	7	3	3	5
**Age (y), mean±SD**	40.2±5.1	52.6±5.0	77.3±3.2	53±13.9	45.6±10.0
**Sex (M/F)**	3/7	5/2	2/1	3/0	2/3
**Eosinophils (/mm** ^**3**^ **), mean±SD**	137±27.37	1256±287.4	2753±587	1577±76.23	2866±1311
**minmax**	60340	5002880	16003520	14601720	11808050

### Eosinophil isolation

**Fig 1:**
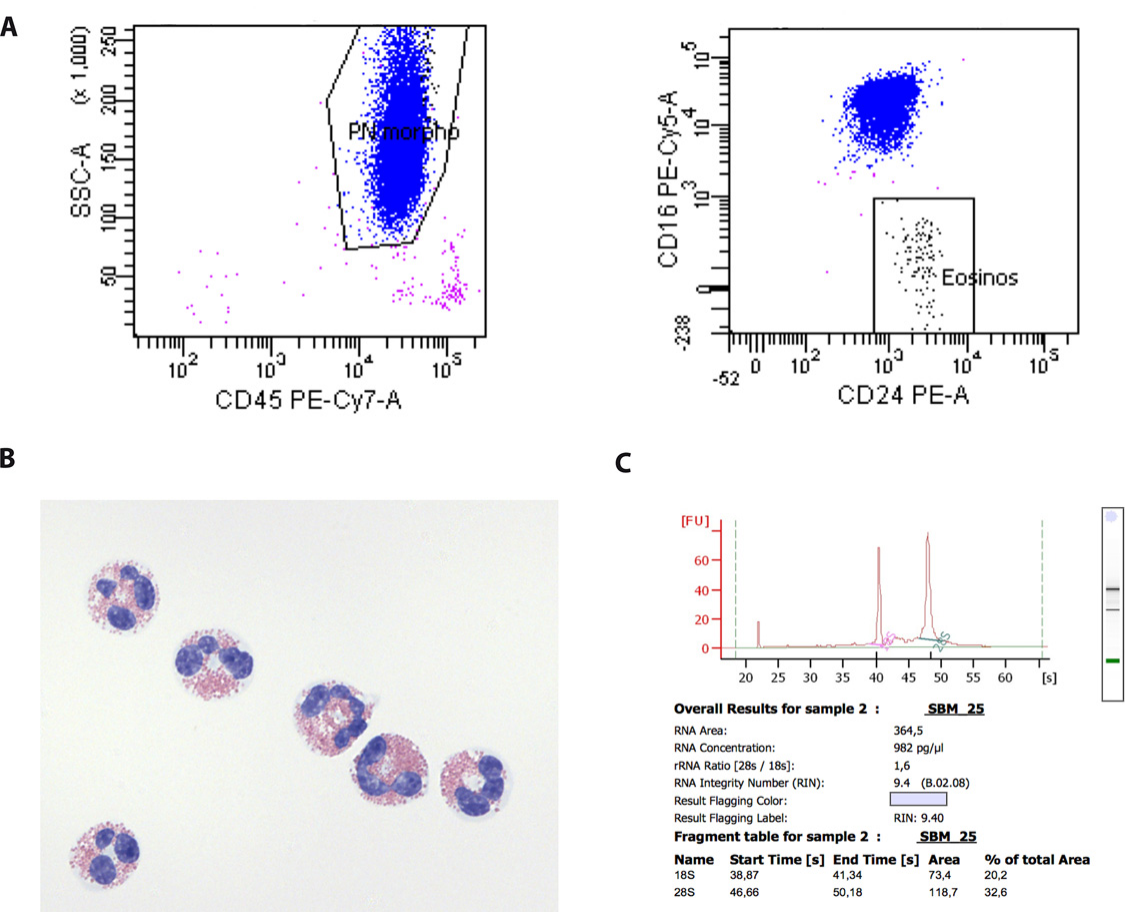
Eosinophil isolation by FACS generates high quality RNA. **(A)** Flow cytometry gating strategy for the identification of eosinophils. Eosinophils were identified among a granulocyte suspension (see Methods) as a CD16 fluorescence negative cell population **(B)** Purity of sorted eosinophils as assessed by cytospin preparation was close to 100%. **(C)** Bioanalyzer RNA profile with RNA integrity number (RIN) of an eosinophil sample. All RNA samples included in the expression analysis had a RIN > 8.

First, granulocytes were isolated by density-gradient centrifugation over Histopaque 1077 and Histopaque 1119 (Sigma Aldrich). Residual erythrocytes were lysed by Versalyse (Beckman Coulter). The granulocyte suspension was stained with anti-CD45-PC7, CD24-PE and CD16-PC5 (Beckman Coulter). Eosinophils were sorted from the granulocyte suspension by FACSAria (BD Biosciences) based on FSC and SSC characteristics, CD45 and CD24 expression and lack of CD16 expression. All steps were performed on ice. Eosinophil viability after FACS sorting was assessed by trypan blue exclusion (>90%) and purity (>99%) was assessed by FACS (data not shown) and cytospin (Fig 1B). Cells were stored in the lysis buffer (see below) at -80°C until batch RNA extraction.

### RNA extraction

Total RNA was extracted from sorted eosinophils using the RNeasy Micro Kit (Qiagen) according to the manufacturer’s instructions. RNA concentration was assessed by a NanoDrop spectrophotometer and RNA quality was measured using an Agilent Bioanalyzer. All RNA samples included in the expression analysis had an RNA integrity number (RIN) > 8 (Fig 1C and S1 Fig).

### Microarrays

Microarray analysis was performed according to Agilent protocol “Two-Color Microarray-Based Gene Expression Analysis–Low Input Quick Amp Labeling” version 6.5, May 2010 (Cat # G4140-90050). Test samples (asthma n = 4, healthy controls n = 3, dermatological disease n = 3, pulmonary aspergillosis n = 4, parasitosis n = 3) were linearly amplified and labeled with Cyanine 3, starting from 25 ng of total RNA. In parallel, the “Human Universal Reference Total RNA” (Clonetech, Cat # 636538) was amplified and labeled with Cyanine 5. Each experimental sample was then co-hybridized with the unique human universal reference on Agilent “SurePrint G3 Human Gene Expression 8x60K Microarray” (Design ID 028004), for 17 hours, at 65°C under 10 rpm. Following washing, the slides were scanned using an Agilent G2565CA microarray Scanner System, at a 3μm resolution in a 20-bit scan mode, according to the “AgilentG3_GX_2Color” protocol. Raw.tiff images were then extracted using Agilent “Feature Extraction, version 10.10.1.1” following “GE2_1010_Sep10” protocol. Median raw expression values were further normalized by quantile method [14]. Differentially expressed (DE) genes were selected using the fold change (FC) based method proposed by Dembélé et al [15]. This method proceeds as follows: using pair of samples from two biological conditions, FCs are computed for all the genes. These FCs are sorted in increasing order and their ranks are associated to genes. This is repeated with all the pair of samples in the dataset. An average rank is computed and used as statistic leading to p-values for genes. Finally a threshold (0.01) is used to select significant genes. Importantly, there is no multiple comparison issue(s) with this method.

### Gene ontology analyses

Functional enrichment was assessed using the DAVID database http://david.abcc.ncifcrf.gov/.

### Real-time quantitative Reverse Transcription PCR (RT-qPCR)

Total RNA was reverse transcribed using the iScript cDNA Synthesis Kit according to the manufacturer’s instructions (BioRad). Quantitative PCR was performed in a total volume of 20 μl using SsoAdvanced^TM^ SYBR Green Supermix kit (BioRad) and gene-specific primers: *IL-8* 3’ AGCACTCCTTGGCAAAACTG, *IL-8* 5’ CGGAAGGAACCATCTCACTG; *AQP9* 3’ TGCAACTGCCATTGAAAATC, *AQP9* 5’ AGTTCTTGGGCACGTTCATC; *IL-2RA* 3’ TAGGCCATGGCTTTGAATGT, *IL-2RA* 5’ ATACCTGCTGATGTGGGGAC; *LIPA* 3’ GCAAGGTTTGTGACCCAGTT, *LIPA* 5’ TGCCTTAACCGAATTCCTCA; *IL-10RA* 3’ GGTTCACACTGCCAACTGTC, *IL-10RA* 5’ ACCTTACCGCAGTGACCTTG; *PMP22* 3’ TTGGCAGAAGAACAGGAACA, *PMP22* 5’ CCTCAGGAAATGTCCACCAC; *CD83* 3’ GGGGTGTCTCCATCCTCTCT, *CD83* 5’ TGCTCCGAAGATGTGGACTT; *CCL3* 3’ TGGCTGCTCGTCTCAAAGTA, *CCL3* 5’ TGCAACCAGTTCTCTGCATC. After an initial denaturation step at 96° C for 10 min, the cycling conditions were 95° C for 10 s/60° C for 30 s, using a Rotor-Gene™ 6000 real-time PCR machine (Corbett Life Science). Amplification products were detected as an increased fluorescent signal of SYBR^®^Green during the amplification cycles. Results were obtained using Rotor-Gene 6000 Series Software and evaluated using Excel (Microsoft). Melting-curve analysis was performed to assess the specificity of PCR products. Results were normalized to *β-actin* levels. The RT-qPCR results are presented as means and standard error of the mean (SEM). Differences in the expression of mRNA were determined by using a two-tailed non parametric Mann-Whitney test with the GraphPad Prism 6 software. P values <0.05 were considered as significant.

## Results

### Isolation of eosinophils by fluorescence activated cell sorting (FACS) yields high quality RNA

Eosinophils were efficiently isolated from human peripheral blood by FACS and identified as a CD16 fluorescence negative cell population in an enriched granulocyte suspension (see Methods and Fig 1A). Eosinophils were kept at 4°C during the whole isolation process to block cell activation. The purity of the sorted eosinophil population was close to 100%, as assessed by FACS and cytoslides (Fig 1B). Using this isolation procedure, high quality RNA was obtained from hypereosinophilic subjects and from healthy controls with RNA Integrity Numbers (RIN) > 8. A total amount of around 50 ng of total RNA was obtained from each sample (Fig 1C and S1 Fig).

### Expression profiles of circulating eosinophils differ between asthmatic and healthy subjects

**Fig 2:**
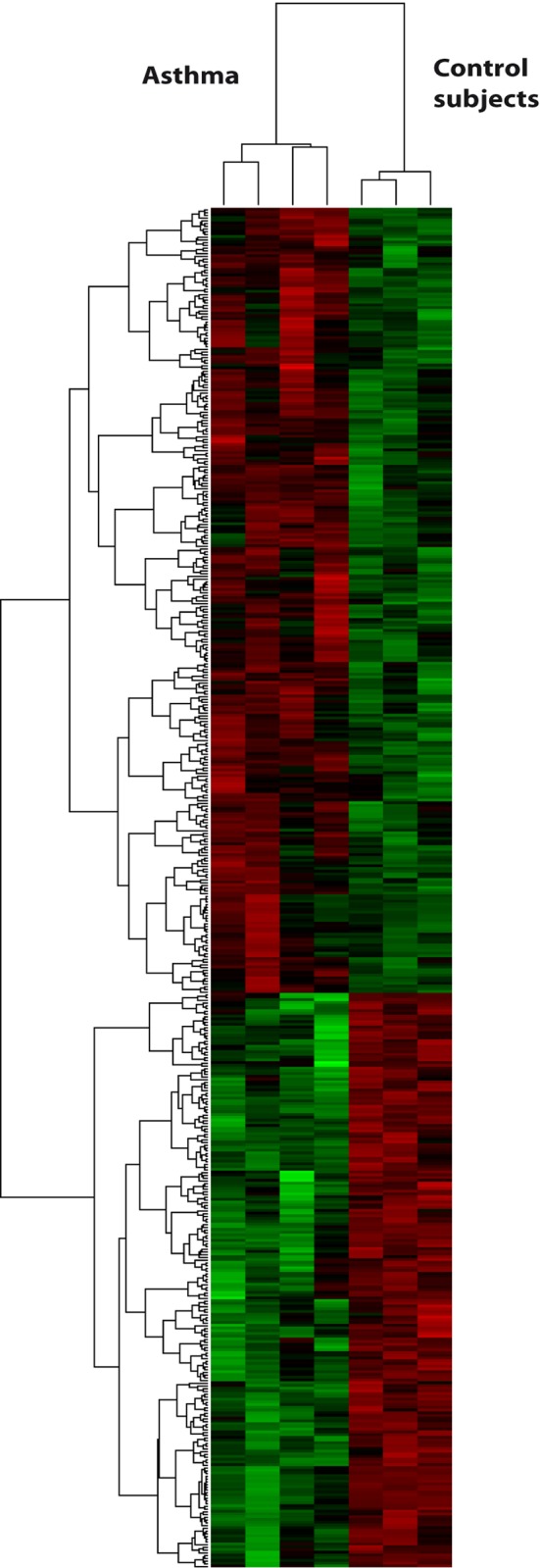
Circulating eosinophils in asthma differ in their gene expression profile when compared to healthy subjects. Heat map of hierarchical clustering of the top expressed genes of circulating eosinophils from subjects with asthma (*n* = 4) *vs* healthy controls (*n* = 3). The horizontal dendrogram represents the relationship between asthmatic and healthy subjects. The vertical dendrogram represents the relationship between the expression levels of each gene across all the samples. Over-expressed genes are shown in red and under-expressed genes are depicted in green.

For patient characteristics see Materials and Methods. Complete gene expression profiles from peripheral eosinophils were generated from asthmatic patients (n = 4) and non-atopic healthy controls (n = 3) upon RNA hybridization to Agilent microarrays measuring the expression levels of 27,958 Entrez Genes. Supervised hierarchical clustering analysis of the top over- and under- expressed genes (Fig 2) and unsupervised hierarchical clustering (S2 and S3 Figs) enabled us to discriminate asthmatic subjects from healthy controls. Statistical analysis [15] identified a total of 47 genes displaying a 2-fold or greater (21 up-regulated, 26 down-regulated) variation in eosinophils of asthmatic subjects when compared to healthy controls.

Table 2 shows the list of genes with significant mRNA expression fold changes in circulating eosinophils of patients with asthma compared to healthy controls. Remarkably, several of the transcripts exhibiting significant up- or down-modulation—i.e, *IL-2RA*, *IL-3RA*, *GSTT1*, *EGR1*, *EGR2*, *PMP22*, *IL-8*—have been reported in previous studies to be regulated in blood eosinophils in response to *in vitro* exposure to *IL-5* or granulocyte macrophage-colony-stimulating factor (*GM-CSF*), cytokines considered as the most active triggers of eosinophil activation [16] [17] [18]. An overview of the function of these 47 genes suggests that most of them exhibit immune-related functions (Table 2). Indeed, the most strongly up-regulated (20.33 fold) gene was *DEFB105B*, an effector component of the innate immune system supposed to have broad antimicrobial activity [19]. The most strongly down-regulated genes also encode immune-related genes; HLA class II, -11.38 fold and the *TLR1*, -5.47 fold. In addition, Prostaglandin-endoperoxide synthase 2 (*PTGS2*), a key enzyme for pro-inflammatory prostaglandin production and IL-8, a major pro-inflammatory cytokine, were also down-regulated (-2.95 and -2.32 fold respectively). *CHRM4* a receptor for acetylcholine was up regulated (2.05 fold), thus confirming recent findings suggesting that acetylcholine can regulate functions in the respiratory tract, including inflammation and remodeling during inflammatory lung diseases [20]. Other genes encode cell surface proteins (i.e., *IL-2 receptor alpha subunit (RA)*, *IL-3RA*, *IL-10RA*), intracellular enzymes related to neutralization of oxidative stress (*GSTT1*) [21], transcription factors (*EGR1*, *EGR2*) and proteins of unknown function (*PMP22*).

**Table 2:** Genes significantly up- or down-regulated in circulating eosinophils of patients with asthma compared to healthy controls.

Accession No. ^§^	Gene Symbol	Gene Name	Fold change
NM_001040703	DEFB105B	defensin, beta 105B	20.33
NM_000709	BCKDHA	branched chain keto acid dehydrogenase E1, alpha polypeptide	10.48
NM_002201	ISG20	interferon stimulated exonuclease gene	4.69
NM_015515	KRT23	keratin 23 (histone deacetylase inducible)	4.68
NM_019086	VSIG10	V-set and immunoglobulin domain containing 10	3.14
NM_032607	CREB3L3	cAMP responsive element binding protein 3-like 3	3.04
NM_000417	IL2RA^#^	interleukin 2 receptor, alpha	2.92
NM_001558	IL10RA^#^	interleukin 10 receptor, alpha	2.62
NM_002183	IL3RA	interleukin 3 receptor, alpha (low affinity)	2.53
ENST00000367534	ENST00000367534	Actin-related protein 2/3 complex subunit 5 (Arp2/3 complex 16 kDa subunit)(p16-ARC)	2.34
NM_000304	PMP22^#^	peripheral myelin protein 22	2.28
NM_000853	GSTT1	glutathione S-transferase theta 1	2.22
NM_014020	TMEM176B	transmembrane protein 176B	2.21
NM_005125	CCS	copper chaperone for superoxide dismutase	2.18
NM_002966	S100A10	S100 calcium binding protein A10	2.17
NM_152331	ACOT4	acyl-CoA thioesterase 4	2.16
NM_000235	LIPA^#^	lipase A, lysosomal acid, cholesterol esterase	2.16
NM_020879	CCDC146	coiled-coil domain containing 146	2.14
ENST00000449914	ENST00000449914	Unknown	2.10
NM_003806	HRK	harakiri, BCL2 interacting protein (contains only BH3 domain)	2.09
NM_000741	CHRM4	cholinergic receptor, muscarinic 4	2.05
ENST00000399670	ENST00000399670	HLA class II histocompatibility antigen, DQ(1) beta chain Precursor (DC-3 beta chain)	-11.38
NM_003263	TLR1	toll-like receptor 1	-5.47
NM_002123	HLA-DQB1	major histocompatibility complex, class II, DQ beta 1	-4.58
NM_006167	NKX3-1	NK3 homeobox 1	-4.26
NR_003937	HLA-DQB2	major histocompatibility complex, class II, DQ beta 2	-3.49
NM_002982	CCL2	chemokine (C-C motif) ligand 2	-3.34
NM_005217	DEFA3	defensin, alpha 3, neutrophil-specific	-3.32
NM_032882	PNMA6A	paraneoplastic antigen like 6A	-3.30
NM_000963	PTGS2	prostaglandin-endoperoxide synthase 2 (prostaglandin G/H synthase and cyclooxygenase)	-2.95
NM_052926	PNMA5	paraneoplastic antigen like 5	-2.88
NM_020873	LRRN1	leucine rich repeat neuronal 1	-2.42
NM_021983	HLA-DRB4	major histocompatibility complex, class II, DR beta 4	-2.39
NM_020980	AQP9^#^	aquaporin 9	-2.35
NM_000399	EGR2	early growth response 2	-2.35
NM_002983	CCL3^#^	chemokine (C-C motif) ligand 3	-2.34
NM_000584	IL8^#^	interleukin 8	-2.32
NM_001964	EGR1	early growth response 1	-2.32
NM_133271	FCAR	Fc fragment of IgA, receptor for transcript variant 3	-2.31
NM_001925	DEFA4	defensin, alpha 4, corticostatin	-2.31
NM_001772	CD33	CD33 molecule, transcript variant 1	-2.29
ENST00000366784	ENST00000366784	Inositol-trisphosphate 3-kinase B (EC 2.7.1.127) (IP3 3-kinase B)(IP3K-B)(IP3K B)	-2.28
NM_001523	HAS1	hyaluronan synthase 1	-2.21
NM_138813	ATP8B3	ATPase, class I, type 8B, member 3	-2.20
NM_173198	NR4A3	nuclear receptor subfamily 4, group A, member 3, transcript variant 2	-2.12
NM_004665	VNN2	vanin 2, transcript variant 1	-2.09
NM_004233	CD83^#^	CD83 molecule	-2.04

§: GenBank accession numbers

#: genes chosen for validation by RT-qPCR.

To get a broader view of these differentially expressed genes, gene ontology analysis was performed using the DAVID database (Database for Annotation, Visualization and Integrated Discovery) [22]. The statistically significant (p<0.01) functional categories (filtered to exhibit at least 10% concordance), which were enriched, are predominantly related to response to stimuli (i.e., external, organic, biotic, chemical), homeostatic processes, response to wounding and inflammatory responses (Table 3). Incidentally, this analysis also reveals a linkage between this set of genes and asthma (p = 0.006) among few other diseases in this list (Table 3).

**Table 3:** Gene ontology categories with an enrichment score p<0.01 and > 10% of gene concordance identified using the DAVID database.

Gene ontology category	*p*	Concordance (%)
glycosylation site:N-linked (GlcNAc. . .)	0.003	38,3
glycoprotein	0.004	38,3
topological domain:Cytoplasmic	0.004	34,
topological domain:Extracellular	0.001	31,9
molecular transducer activity	0.003	25,5
signal transducer activity	0.003	25,5
plasma membrane part	0.009	25,5
response to chemical stimulus	0.0004	23,4
response to external stimulus	0.0001	21,2
receptor activity	0.009	21,2
response to organic substance	0.0001	19,1
response to biotic stimulus	0.0002	14,8
behavior	0.0006	14,8
Immunoglobulin-like fold	0.001	14,8
multi-organism process	0.004	14,8
homeostatic process	0.007	14,8
regulation of cell proliferation	0.008	14,8
response to other organism	0.0005	12,7
inflammatory response	0.0008	12,7
negative regulation of cell proliferation	0.001	12,7
Cytokine-cytokine receptor interaction	0.002	12,7
multiple sclerosis	0.002	12,7
cell motion	0.004	12,7
Immunoglobulin-like	0.005	12,7
asthma	0.006	12,7
response to wounding	0.007	12,7
sarcoidosis	0.0001	10,6
response to bacterium	0.001	10,6
stomach cancer	0.002	10,6
anatomical structure formation involved in morphogenesis	0.008	10,6
response to hormone stimulus	0.010	10,6

**Fig 3:**
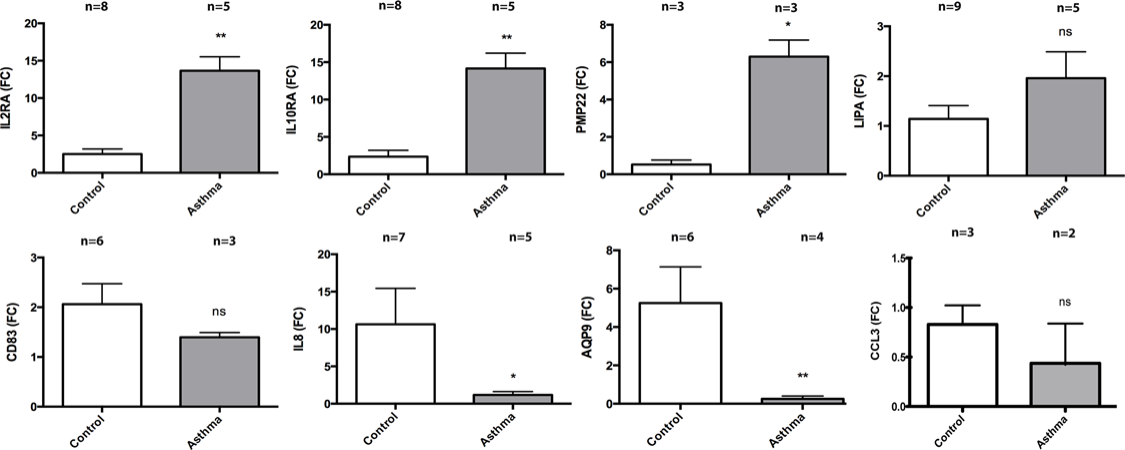
RT-qPCR of selected genes. Gene expression was determined by RT-qPCR in eosinophils isolated from subjects with asthma and healthy controls. Results were normalized to *β-actin* and expressed as fold change compared with samples from healthy controls. Results are presented as means and SEM. *p<0.05; **p<0.01 (2-tailed non parametric Mann-Whitney).

The above described microarray observations were validated by quantitative RT-PCR (RT-qPCR) for 8 of the 47 differentially expressed genes. Results were obtained from remaining RNA samples (n = 2) used in the microarray analysis and from newly obtained eosinophil RNA samples (Fig 3). Despite differences that did not reach statistical significance in the case of *CD83*, *CCL3* and *LIPA*, gene expression modulation in eosinophils from asthmatic patients is statistically (p = 0.0279, Spearman r = 0.7857) similar to our observation using microarrays, as evidenced by a correlation analysis between the two sets of data (fold changes obtained with Agilent microarrays *vs*. fold change calculated by RT-qPCR, data not shown).

### Hypereosinophilic disorders display a similar transcriptional profile to asthma

**Fig 4:**
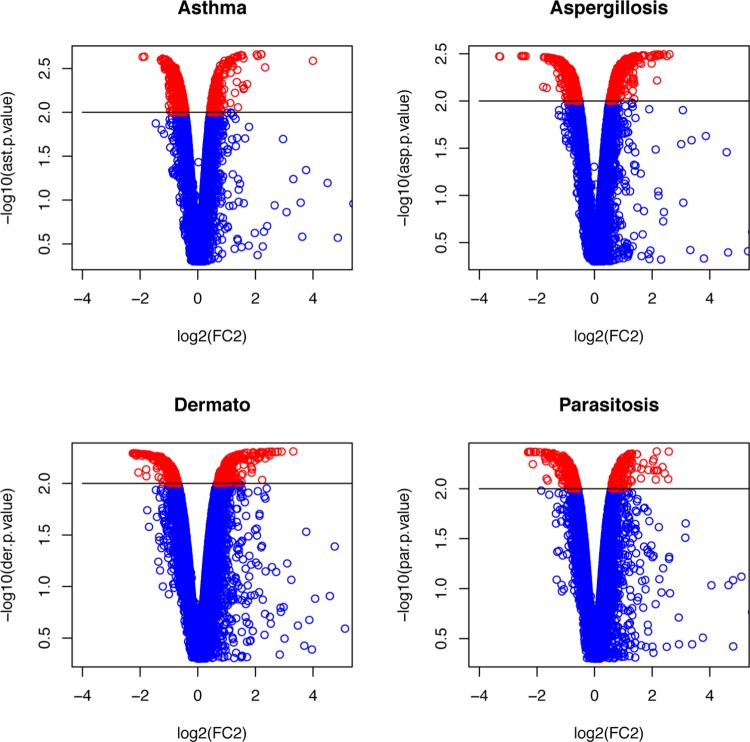
Differentially expressed genes in unrelated hypereosinophilic diseases. Volcano plot of genes differentially expressed between subjects with peripheral hypereosinophilia (i.e. asthma, dermatological disease, parasitosis and pulmonary aspergillosis) *vs*. healthy controls. Genes with a p-value <0.01 are depicted in red.

Next, we checked whether these differentially expressed genes were specific to asthma. To this end, we first isolated peripheral blood eosinophils from patients with other hypereosinophilic disorders, including subjects with peripheral eosinophilia related to dermatological diseases, parasitosis and pulmonary aspergillosis (see Table 1 for the detailed characteristics of patients) and realized similar microarray-based analysis of their transcriptome. As in case of asthmatic eosinophils versus those of healthy controls, the transcriptomic profile in eosinophils isolated from other hypereosinophilic conditions was also distinct from that of healthy subjects as revealed by volcano plots (Fig 4); highlighting only genes with a statistically significant (p<0.01) fold change (red dots). In addition, this representation also indicates that many genes exhibit large, potentially pathophysiologically-relevant changes in their transcriptional profile. But the p-value for these modifications in expression does not reach statistical significance, and the corresponding genes are therefore excluded from the following analyses. These variations likely reflect, at the molecular level, the inherent heterogeneity of our samples which is phenotypically evidenced by the clinical variations described in Table 1.

To obtain an overview of the genes differentially expressed in the different diseases, we first selected those showing a statistically significant (p<0.01), higher or lower, expression level with a minimum fold change (FC) >+/- 2 as compared to healthy subjects (S1 Table). The comparison of these lists shows that only 23 to 37% (13/47 in asthma; 23/100 is aspergillosis; 61/163 in dermatological diseases and 30/87 in parasitosis) of genes were specifically modulated in each of the four conditions (S4 Fig; S2 Table). Most of the transcriptional changes that occur in patients’ eosinophils are unexpectedly common: 10 genes are common to the 4 diseases, and 40 are found in 3 out of 4 of them, indicating a cross-disease transcriptional signature. To confirm and quantify this observation, we performed a correlation analysis of the FC for each gene showing significant (p<0.01) differential expression in at least 1 condition (listed in S2 Table). For each combination of disease tested, correlation (using a Spearman non-parametric assay) appeared to be highly (p<0.0001) significant, which confirms the important similarities between the transcriptional profiles determined for each condition.

**Fig 5:**
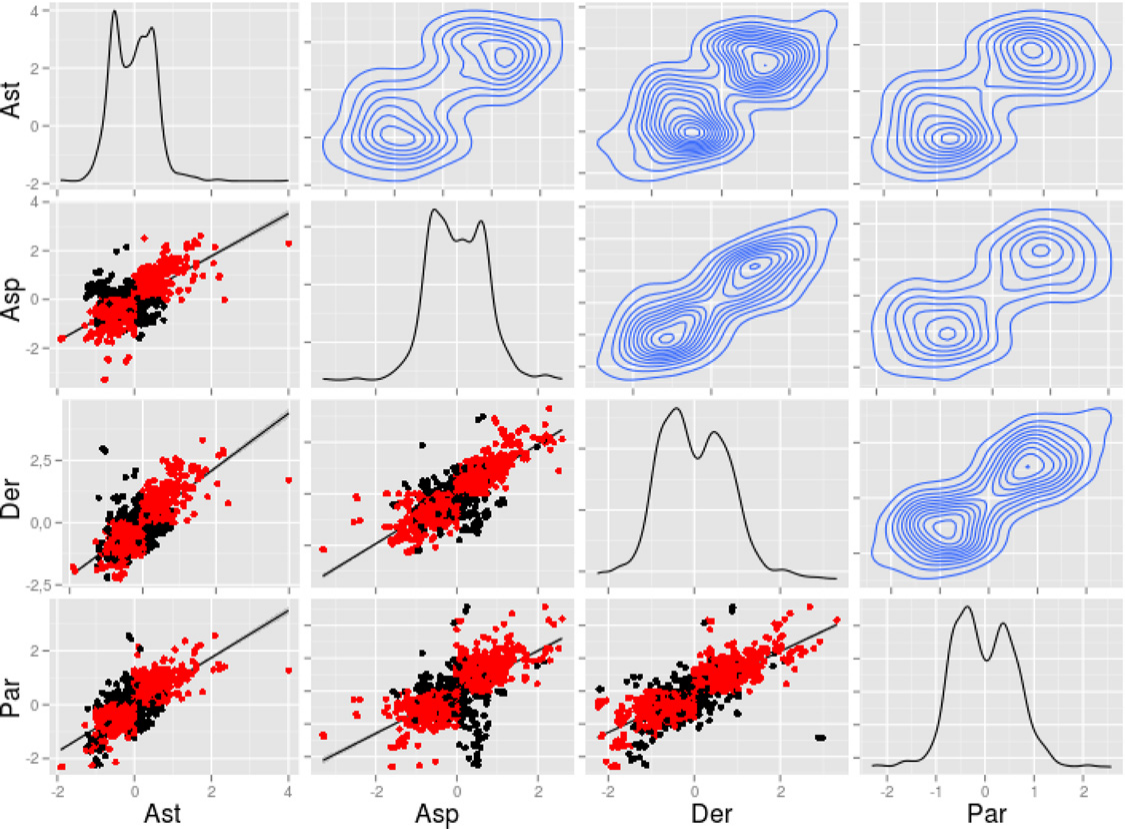
Eosinophils from asthmatic subjects display a similar transcriptional profile as eosinophils from other hypereosinophilic conditions. Scatterplots are based on fold changes of selected genes that are differentially expressed at least in one hypereosinophilic disease (p< 0.01). Diagonal: estimation of the density of log fold changes (lfc) for each variable. Upper elements: estimation of the bivariate density of each couple of variables. In red: genes with a lfc changed to the same direction in all the conditions. In black: genes with a lfc changed to the opposite direction in at least one couple of variables. With a cut-off for the lfc of 0.15, more than 95% of the probe sets have identical changes. Ast, asthma; Asp, pulmonary aspergillosis; Der, dermatological disease; Par, parasitosis.

Finally, using the complete list of genes with significant (p<0.01) fold change in at least one of the diseases, we estimated the distribution of the log fold changes (lfc) in each disease group. As expected, two peaks appear in the univariate analysis, corresponding to the groups of up- and down-regulated genes, respectively (Fig 5). The bivariate analysis indicates that the modulation of gene expression is very similar between two diseases, as revealed by (i) the fact the genes which are characterized by opposite transcriptional profiles in one condition out of four (depicted in black) are in minority compared to those which are modulated in the same direction in the four diseases (in red); and (ii) the very homogenous shape of the estimated density plots between each couple of diseases. This analysis confirmed highly identical changes with more than 95% of the regulated genes being similar in all conditions. Analyzing some individual genes, we additionally observed that transcriptional changes of outliers (i.e. NR4A3 with a lfc > 2 in the dermatological disease group and a lfc < -1 in parasitosis group) were not statistically significant.

## Discussion

Eosinophils circulate in low numbers in the peripheral blood in healthy individuals and their detailed analysis has been limited by the difficulty to obtain pure cells in large amounts. Conventional methods for eosinophil purification use immunomagnetic cell separation and require a large (120 ml) blood volume [23]. Moreover, eosinophils contain proteins with potent ribonuclease activities, making RNA isolation challenging. For the present study, we setup an efficient FACS-based protocol for isolating intact human eosinophils from peripheral blood. Only small (20 ml) volumes of whole blood were necessary for eosinophil purification and subsequent high quality RNA extraction, even from healthy subjects generally exhibiting low levels of circulating eosinophils.

The subsequent transcriptomics analysis by microarray of a pure isolated eosinophil population, which, to our knowledge has not been realized so far in such setting, compared healthy controls to asthmatic patients with peripheral hypereosinophilia. Indeed, whilst other groups have previously established transcriptome signatures between healthy controls and other hypereosinophilic diseases (i.e. eosinophilic esophagitis) this has been done in tissue samples obtained by biopsies which by nature contain other (non-eosinophil) cellular elements [24, 25].

We identified 47 genes significantly modulated by at least 2-fold in asthmatic subjects. This relatively small number of differentially expressed transcripts might reflect a poor plasticity of this leukocytes subset when compared for example to CD8^+^ T cells in which 1566 differentially regulated genes have been identified in severe asthma [26]. Interestingly, we noted that some of the differentially expressed genes identified in the course of this study were already shown to be regulated in eosinophils, thereby further validating our microarray data. For instance, expression of *IL-2RA*, *IL-3RA*, *GSTT1*, *EGR1*, *EGR2*, *PMP22* and *IL-8* have been previously reported to be regulated in blood eosinophils in response to *in vitro* exposure to IL-5 or granulocyte macrophage-colony-stimulating factor (GM-CSF) [18] [27] [16], two important growth and maturation factors for eosinophils which stimulate their effector functions [1].

A first potential limit to these findings is that purified eosinophils may not completely and accurately reflect the *in vivo* situation because of cell activation that could result from the isolation procedure. However, in our hands, the expression of the eosinophil activation markers *CD69* or L-selectin (*CD62L*) between patients and healthy subjects remains constant, as it is the case for unfractionated blood eosinophils [28]. Second, eosinophils are tissue-dwelling cells and depending on their location and the microenvironment, their activation status might differ [1]. While we cannot presently object to this caveat, we wish to stress that our transcriptomic-based approach identified numerous genes critical in eosinophils function and therefore, provides the foundations for further expression studies using RT-qPCR aiming at selectively differentiating circulating from resident cells. We were also unable to show a significant regulation of some of already described eosinophil adhesion molecules such as integrins which expression might be increased in inflammatory conditions [1]. Finally, our relatively small sample size might have impeded the detection of all significantly regulated genes between health and disease.

Traditionally, eosinophils are considered in disease as activated cells exhibiting destructive capabilities through the release of cytotoxic and pro-inflammatory mediators and are linked in many mouse models of allergic respiratory inflammation and human asthma studies to the underlying pulmonary pathology [3, 29]. However, conflicting data have emerged, as recent murine studies using complex genetic asthma models failed to support a significant role for eosinophils in inflammatory tissue damage [7] [30]. Moreover, improvement of symptoms in asthma patients after therapies targeting eosinophils have not been linear and have encountered limited success, even if eosinophil numbers are dramatically reduced in the blood and airways following these therapies [4] [31] [5, 32] [33] [34]. In line with these observations, our data indicate that cytotoxic markers such as granule proteins or cysteinyl leukotrienes are not differentially expressed in asthmatic eosinophils vs controls, at least in peripheral eosinophils. Furthermore, *prostaglandin-endoperoxide synthase 2*, a key enzyme in the production of pro-inflammatory prostaglandins [35], and *IL-8*, a major pro-inflammatory cytokine [36], were also down-regulated, suggesting that increased cytotoxic or pro-inflammatory actions are not the hallmarks of circulating eosinophils in asthma.

*DEFB105B*, an alarmin, belonging to the beta-defensin family, was strongly up regulated in asthmatic eosinophils. Recently, various defensins have been found to mediate tissue repair and homeostasis. Indeed, human *DEFB3* over-expression through viral transduction promotes skin wound healing in a pig model [37]. *S100A10*, also known as *p11*, is another alarmin gene which exhibits up-regulation in asthmatic eosinophils in our study. *S100A10* is a well-described binding partner of annexin A2, which is thought to play an anti-inflammatory role [38, 39]. S100A10 can also inhibit phospholipase A2 activity *in vitro* [40], a key enzyme for pro-inflammatory leukotriene, prostaglandine and platelet activating factor biosynthesis. In addition, our data indicate that *LIPA*, also known as cholesterol ester hydrolase, was up-regulated in eosinophils from asthmatic patients. LIPA plays a central role in the modulation of neutral lipid metabolites in all cells [41]. Knockout mice for this gene develop severe abnormal cell proliferation and alveolar remodeling in the lung [42], relating LIPA to pulmonary homeostasis and remodeling. These examples nicely support the current notion claiming that eosinophils, besides their well-established damaging capabilities, might also contribute to heal tissues injured during inflammatory reactions.

The accumulation and increase of eosinophils are not unique to asthma and helminth infections. Eosinophil proliferation is a common feature of numerous other unrelated inflammatory disorders, such as adverse drug reactions, atopic dermatitis, Churg-Strauss syndrome, some malignancies or yet eosinophilic gastrointestinal disorders [1]. Our data didn’t support a disease-specific transcriptional signature in circulating eosinophils from different hypereosinophilic conditions. Surprisingly, a large number of genes were shared between eosinophils from asthmatic subjects and eosinophils from other hypereosinophilic diseases. This underlines again the non-specific role of circulating eosinophils in asthma and other hypereosinophilic diseases and identifies this cell as a major secondary recruited cell in an ongoing inflammatory process.

In conclusion, this is the first report of genome-wide transcriptomic analysis of human peripheral blood eosinophils isolated from individual patients and healthy controls by flow cytometry. A disease-specific transcriptional signature could not been identified (one has, however, to bear in mind the small sample size) but our results clearly support new non disease specific roles for eosinophils in pathophysiology, as being major non-specific immunodulators recruited to inflammatory sites and participating in tissue repair and maintenance of homeostasis.

## Supporting Information

S1 Fig Bioanalyzer profiles of total RNA prepared from eosinophil samples used for this study. Eosinophil isolation by FACS generated high quality RNA with RNA integrity number (RIN) > 8 for all the samples.
(TIF)

S2 Fig Heat map of unsupervised hierarchical clustering of the genes in human circulating eosinophils from subjects with asthma (*n* = 4) *vs* healthy controls (*n* = 3). The horizontal dendrogram represents the relationship between asthmatic and healthy subjects.
(TIF)

S3 Fig Principal Component Analysis plot of asthma samples (Asth) and healthy controls (Ctrl). (TIF)

S4 Fig Venn diagram of differentially-expressed genes in the different diseases. Genes with a significant (p<0.01) fold change (FC) >+/-2 were selected.
(TIF)

S1 Table List of all candidate genes in asthma; pulmonary aspergillosis; dermatological disease and parasitosis. (XLSX)

S2 Table Detailed list of exclusive and shared genes in the Venn Diagram (see S4 Fig). (XLSX)
