# Supplementary figures and images for: Circulating Human Eosinophils Share a Similar Transcriptional Profile in Asthma and Other Hypereosinophilic Disorders
Source: PLoS One. 2015 Nov 2;10(11):e0141740. doi: 10.1371/journal.pone.0141740 (PMC4629890; doi:10.1371/journal.pone.0141740)

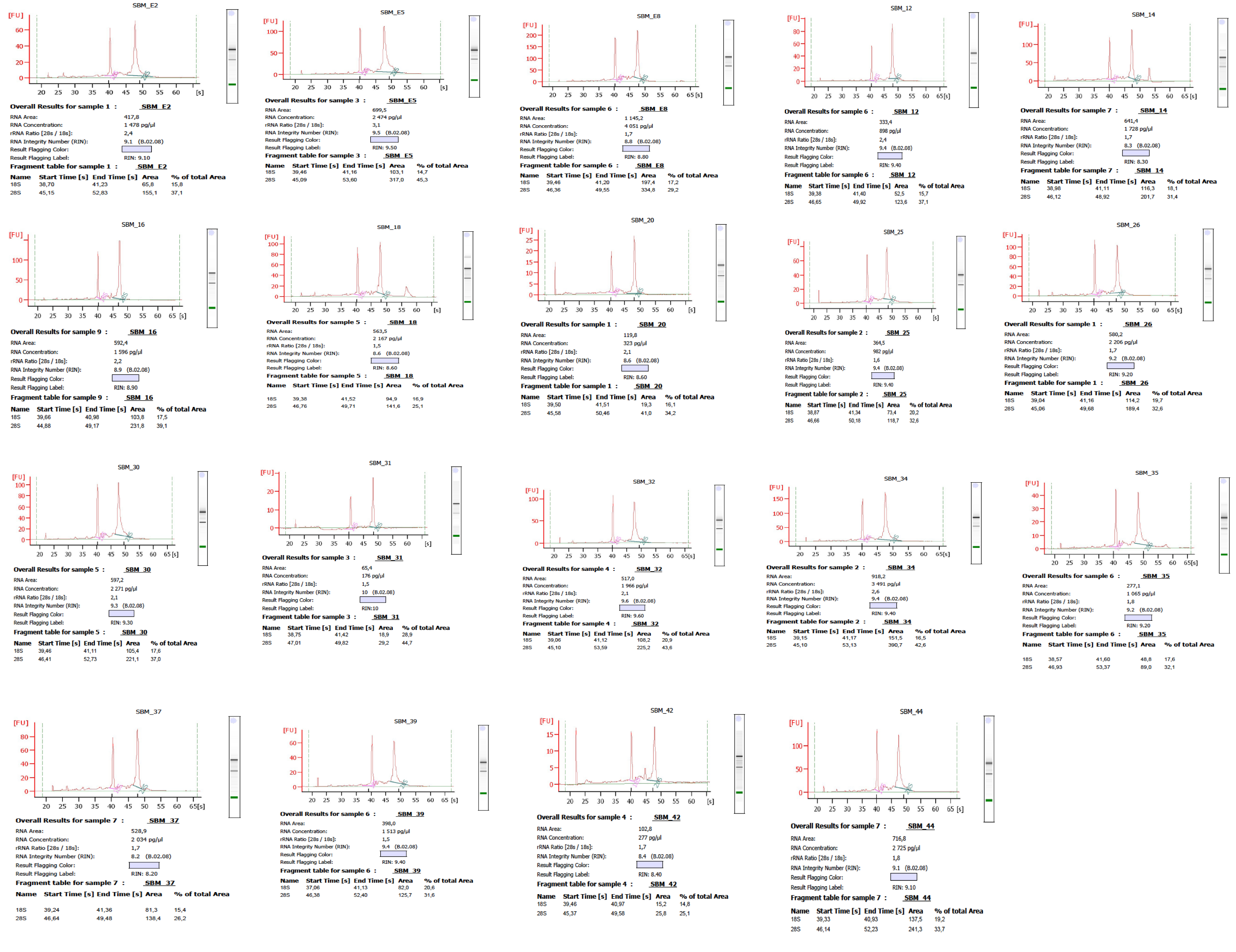

Supplement: S1 Fig — Eosinophil isolation by FACS generated high quality RNA with RNA integrity number (RIN) > 8 for all the samples. (TIF) [file pone.0141740.s001.tif]

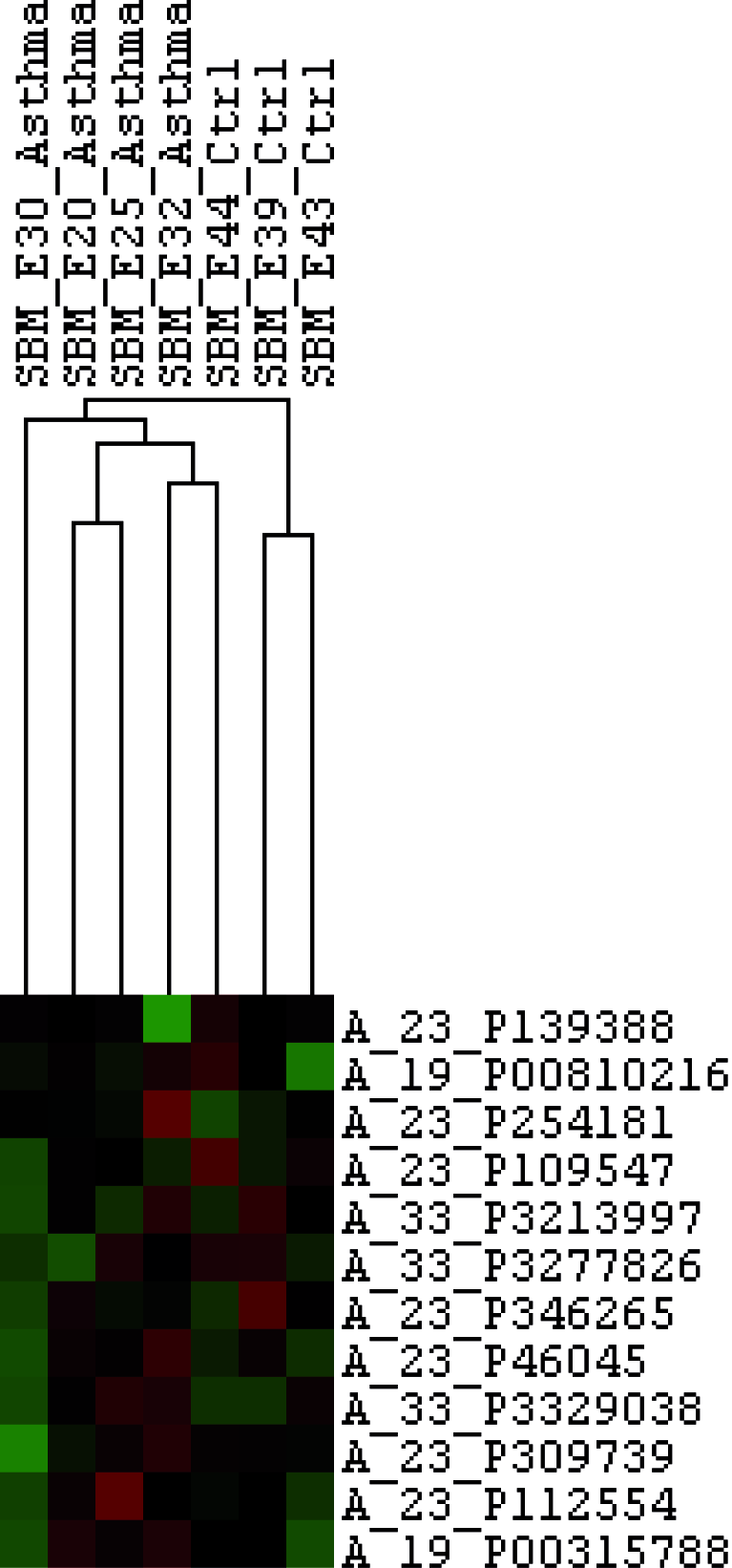

Supplement: S2 Fig — The horizontal dendrogram represents the relationship between asthmatic and healthy subjects. (TIF) [file pone.0141740.s002.tif]

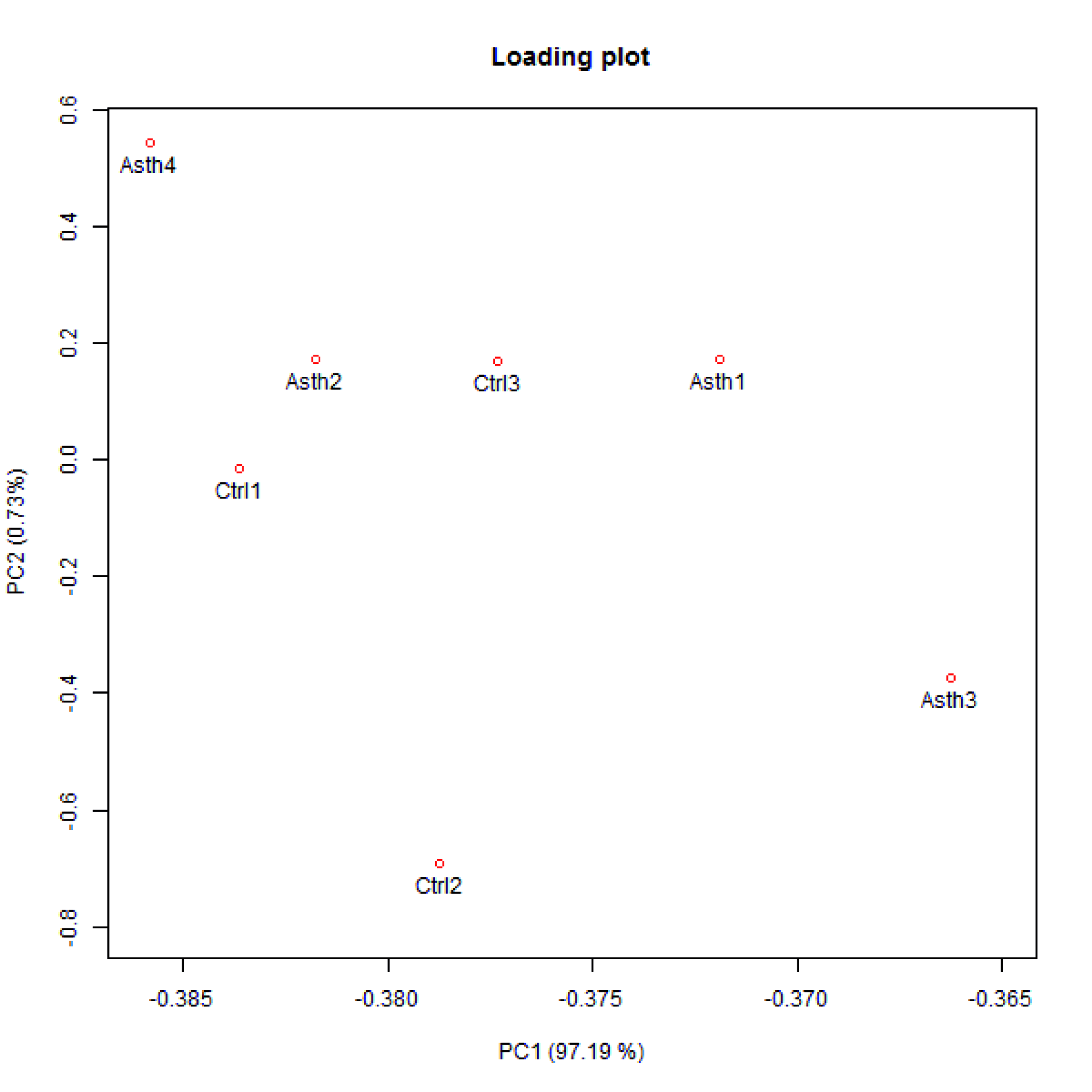

Supplement: S3 Fig — (TIF) [file pone.0141740.s003.tif]

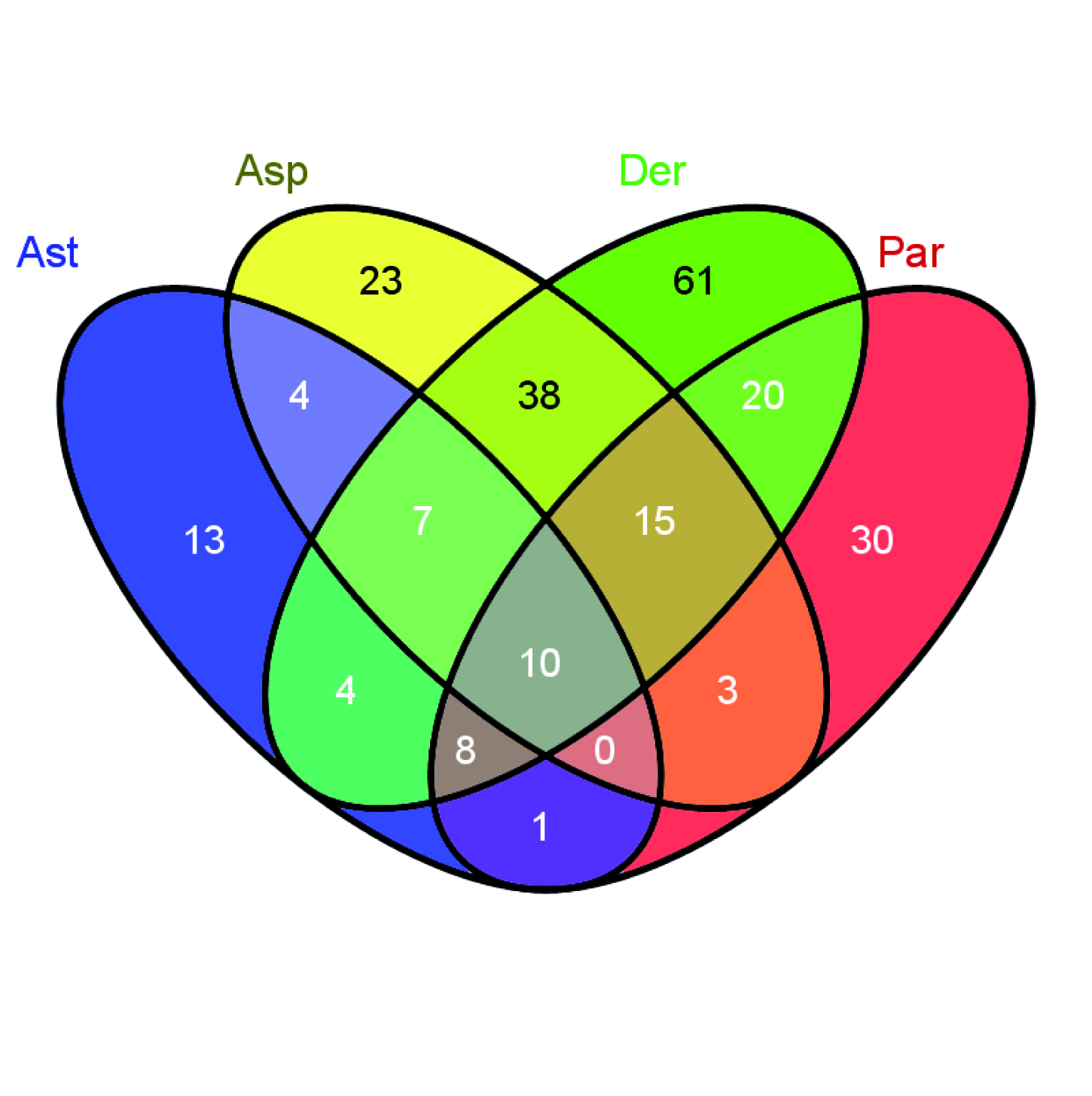

Supplement: S4 Fig — Genes with a significant (p<0.01) fold change (FC) >+/-2 were selected. (TIF) [file pone.0141740.s004.tif]
